# Design of a High Dynamic Range Acquisition System for Airborne VNIR Push-Broom Hyperspectral Camera

**DOI:** 10.3390/s26082474

**Published:** 2026-04-17

**Authors:** Haoyang Feng, Yueming Wang, Daogang He, Changxing Zhang, Chunlai Li

**Affiliations:** 1 Hangzhou Institute for Advanced Study, University of Chinese Academy of Sciences, Hangzhou 310024, China; fenghaoyang23@mails.ucas.ac.cn; 2 Shanghai Institute of Technical Physics, Chinese Academy of Sciences, Shanghai 200083, China; hedaogang@mail.sitp.ac.cn (D.H.); zhangchangxing@mail.sitp.ac.cn (C.Z.); lichunlai@mail.sitp.ac.cn (C.L.); 3 University of Chinese Academy of Sciences, Beijing 100049, China

**Keywords:** airborne hyperspectral, high frame rate, high dynamic range, dual-gain CMOS, FPGA, HDR fusion

## Abstract

Achieving a high frame rate and high dynamic range (HDR) under complex illumination remains a significant challenge for airborne push-broom visible-near-infrared (VNIR) hyperspectral cameras. Problematic scenarios typically include high-contrast scenes, such as ocean whitecaps alongside deep water or concurrently sunlit and shadowed urban surfaces. To address this, a real-time HDR acquisition system based on a dual-gain complementary metal–oxide–semiconductor (CMOS) image sensor is proposed. Specifically, a four-pixel HDR fusion method is developed, utilizing an optical calibration setup to accurately determine the fusion parameters and configure the spectral region of interest (ROI) for reduced data volume. The complete workflow, encompassing spectral–spatial four-pixel binning and piecewise dual-gain fusion, is implemented on a field-programmable gate array (FPGA) using a dual-port RAM-based buffering strategy and a low-latency five-stage pipeline. Experimental results demonstrate a minimal processing latency of 0.0183 ms and a maximum frame rate of 290 frames/s. By extending the output bit depth from 11 to 15 bits, the system achieves a digital dynamic range of the final output of 2.03 × 10^4^:1, representing a 9.58-fold improvement over the original low-gain data. The fused HDR data maintain high linearity and good spectral fidelity, with spectral angle mapper (SAM) values at the 10^−3^ level. Featuring a compact and low-power design, this system provides a practical engineering solution for efficient airborne VNIR hyperspectral acquisition.

## 1. Introduction

An airborne visible–near-infrared (VNIR) push-broom hyperspectral camera captures one-dimensional spatial information and continuous spectral data across the VNIR bands within a single exposure. By performing push-broom scanning along the flight direction, it reconstructs two-dimensional spatial information and forms a data cube [1]. This imaging modality holds significant application value in fields such as industrial inspection [2], water remote sensing [3], crop analysis [4], and environmental monitoring [5]. Complementary metal–oxide–semiconductor (CMOS) image sensors are widely employed as detectors in such hyperspectral systems due to their high quantum efficiency in the VNIR band, high level of circuit integration, and relatively simple hardware implementation [6].

During practical observations, a single field of view often contains targets with markedly different brightness levels. In ocean scenes, for example, whitecaps and foam can exhibit VNIR reflectance of 40–60% [7], whereas the water-leaving signal from clear deep water in the same spectral range is much weaker, with reflectance typically around 0.6–1% [8]. Shadowed regions further increase the contrast between bright and dark areas. A similar situation occurs in urban scenes, where occlusion by high-rise buildings causes sunlit and shaded surfaces to appear at the same time. This often compresses, or even obscures, details in shadowed regions and can therefore affect subsequent spectral inversion [9]. In such high dynamic range (HDR) scenes, the luminance span may reach roughly four orders of magnitude [10]. After spectral dispersion, the reflected light from the same target also varies considerably from one band to another. As a result, the CMOS area-array data obtained in a single exposure contain both strong and weak signals across the spatial and spectral dimensions. To preserve details in both bright and dark regions for most HDR scenes, the acquisition system therefore needs a dynamic range on the order of 10^4^.

In contrast, the typical dynamic range of a conventional linear CMOS image sensor in a single exposure is approximately 60 dB, corresponding to a span of only about 10^3^ [11]. As a result, such sensors struggle to directly accommodate the luminance variation encountered in HDR scenes: strong-signal pixels are prone to saturation, while weak-signal pixels are easily buried in noise. This leads to the loss of both bright and dark details in the two-dimensional spatial image and compromises the completeness of spectral information. To enhance the capability of a CMOS acquisition system to capture both bright and dark region details simultaneously, HDR acquisition methods are commonly employed.

Existing studies on HDR acquisition can generally be classified into three categories. The first expands the dynamic range in the temporal dimension by capturing multiple frames under varying exposure times or gain settings. For instance, Nie et al. [12] utilized low-rank representation, adaptive weights, and guided filtering to fuse low-illumination grayscale images, effectively mitigating noise and preserving shadow and highlight details. Building on this, Tan et al. [13] proposed a multi-ghost-suppression, multi-level fusion framework to reduce ghosting artifacts in dynamic scenes. However, for airborne push-broom hyperspectral cameras, multi-frame fusion remains highly susceptible to motion-induced ghosting and registration errors, while the associated fusion algorithms impose a prohibitive computational burden. The second category enhances dynamic range at the hardware level via optical design or device modification—such as spatial modulation or beam splitting—to acquire multiple responses simultaneously. For example, He et al. [14] developed a DMD-based scheme to obtain multiple luminance responses through optical modulation, while Qiao et al. [15] presented an HDR video reconstruction method utilizing variable-ratio beam splitting across multiple sensors. Although these approaches enable parallel multi-channel acquisition, they significantly increase hardware complexity and physical volume. This limitation is particularly detrimental for hyperspectral cameras, where such modules must be seamlessly integrated with intricate dispersive optics. Compared to the aforementioned approaches, the third category acquires multiple complementary responses within a single exposure and fuses them in real time. This effectively mitigates the artifacts introduced by multi-frame fusion and features a straightforward implementation, making it highly suitable for airborne hyperspectral imaging. The development of this approach has evolved from empirical piecewise fusion to image mapping-based fusion, and ultimately to optical calibration-based image fusion. Along this line, He et al. [16] proposed a three-segment HDR synthesis method for dual-channel scientific CMOS data. Although this method successfully extends the dynamic range, its fusion parameters are determined solely by ideal gain ratios rather than through rigorous optical calibration, thereby compromising the stringent spectral consistency required for hyperspectral data. Furthermore, He et al. [17] employed a simplified reverse S-shaped mapping to expand the usable image range prior to multiscale fusion. Although this method derives the fusion formulas and parameters through scientific computation, these parameters rely exclusively on the acquired image data itself, thereby compromising spectral fidelity and hindering subsequent data inversion. More recently, You et al. [18] developed a real-time HDR system for large-format area-array imaging based on a dual-gain CMOS sensor, which operates at 24 frames/s and employs a varying-illumination method for parameter calibration and real-time fusion. However, this approach focuses exclusively on area-array imaging while neglecting dark-end noise performance. The system also incurs a substantial computational latency of 35 ms, which severely restricts the achievable frame rate. Moreover, because its calibration relies heavily on stable light intensity variations, it struggles to guarantee the linearity of the fused data. Beyond the three aforementioned categories, deep-learning-based approaches have also been explored, shifting dynamic-range enhancement to the reconstruction stage [19]. However, these methods rely heavily on the scale and quality of training data, demand substantial computational resources, and remain exceedingly difficult to deploy within high-frame-rate, low-latency acquisition systems. Consequently, these inherent limitations render such data-driven approaches unsuitable for real-time hyperspectral imaging.

To satisfy the aforementioned requirements and provide a reliable solution for real-time HDR hyperspectral data acquisition, this study addresses the inherent limitations of existing single-exposure fusion methods. Specifically, issues such as compromised spectral fidelity, excessive dark-end noise, unrefined optical calibration procedures, and prohibitive computational latency limit the applicability of current approaches to real-time hyperspectral imaging. Consequently, this paper proposes a four-pixel real-time HDR fusion method based on a dual-gain CMOS sensor. To support this approach, an optical calibration scheme is designed to accurately determine the fusion parameters, and a low-latency field-programmable gate array (FPGA) architecture is developed for real-time implementation. The proposed method is intended to preserve spectral fidelity, suppress dark-end noise, and enhance the digital dynamic range of the final output while supporting high-frame-rate operation. Ultimately, this system provides a practical engineering pathway for airborne push-broom VNIR hyperspectral cameras to achieve high-frame-rate HDR information acquisition in complex scenes.

## 2. Methods

### 2.1. Constraint Analysis for Dynamic Range Enhancement

Within the linear operating region of a CMOS sensor, the digital number (DN) value of a single pixel can be approximated as proportional to the equivalent number of photoelectrons accumulated during a single exposure. This relationship is characterized by a linear response with an offset term [20]:

(1) $$D=kN_{e}+b,$$

where D is the pixel DN value, k is the conversion coefficient from the equivalent number of photoelectrons to the pixel DN, $N_{e}$ is the equivalent number of accumulated photoelectrons during the exposure, and b is the offset term.

In the single-exposure, single-gain acquisition mode, once the gain setting is determined, k and b can be approximately regarded as constants. For representative pixels selected from the bright end and dark regions within the same frame of data, let the equivalent numbers of photoelectrons corresponding to their pixel digital outputs be $N_{\mathrm{eb}}$ and $N_{\mathrm{ed}}$, respectively. Then the bright-dark span of this frame can be expressed as

(2) $$C=\frac{N_{\mathrm{eb}}}{N_{\mathrm{ed}}}=\frac{D_{b}-b}{D_{d}-b},$$

where C is the bright-dark span ratio of the frame, $D_{b}$ is the DN value of the bright-end pixel, and $D_{d}$ is the DN value of the dark-end pixel.

In practical applications, to prevent saturation at the bright end of the CMOS sensor and ensure the preservation of highlight details, it is generally necessary to keep the DN value of the bright-end pixel below the saturation threshold, i.e.,

(3) $$D_{b}\leq D_{\mathrm{sat}},$$

where $D_{\mathrm{sat}}$ denotes the saturation DN value of the CMOS sensor.

Substituting Equation (1) into the above inequality yields

(4) $$kN_{\mathrm{eb}}+b\leq D_{\mathrm{sat}},$$

Accordingly, the equivalent number of photoelectrons accumulated at the bright end during the exposure must satisfy

(5) $$N_{\mathrm{eb}}\leq \frac{D_{\mathrm{sat}}-b}{k},$$

By incorporating Equation (2) into Equation (5), we obtain the following constraint on the equivalent number of photoelectrons at the dark end, which can be expressed as

(6) $$N_{\mathrm{ed}}\leq \frac{D_{\mathrm{sat}}-b}{kC},$$

This condition can be further translated into a constraint on the effective signal amplitude at the dark end in the digital domain:

(7) $$D_{d}-b=\frac{D_{b}-b}{C}\leq \frac{D_{\mathrm{sat}}-b}{C},$$

Equation (7) indicates that, under single-exposure, single-gain acquisition, the effective digital amplitude at the dark end is passively reduced as the frame bright-dark span ratio C increases. When the scene to be captured exhibits a large bright-dark span, C increases accordingly. Even if the CMOS bright end remains within the linear region without saturation, the dark end may still stay in a low-DN range for a long time and be submerged by noise. Attempting to recover usable dark-end information by extending exposure time would cause the bright-end pixels to saturate, resulting in significant loss of highlight details. Moreover, during a single exposure, the gain setting and the conversion coefficient k are fixed, restricting the system to a single gain configuration. If a higher gain is selected, Equations (1) and (5) indicate that the dark-end pixel DN value $D_{d}$ increases, but the maximum allowable accumulated equivalent photoelectrons at the bright end $N_{\mathrm{eb}}$ decreases accordingly, rendering bright-end pixels more susceptible to premature saturation. Conversely, if a lower gain is selected, the headroom at the bright end expands, reducing the likelihood of saturation, but the effective amplitude at the dark end is further compressed, making it harder to exceed the noise threshold. Under the high-frame-rate acquisition constraints imposed by push-broom imaging, the exposure time is limited, and dark-end signal accumulation is difficult to compensate by simply extending the exposure. This limitation exacerbates the inherent trade-off, rendering it even more challenging to balance bright- and dark-end performance. Consequently, single-exposure, single-gain acquisition inevitably involves a trade-off in scenes where bright and dark regions coexist.

To enable a consistent system-level evaluation of the final digital output and the dynamic-range extension achieved by the proposed system, this study adopts the digital dynamic range of the final output as the evaluation metric. This relationship is formulated as follows [21]:

(8) $$R_{D}=\frac{D_{\mathrm{sat}}-b}{\delta_{d}},$$

Specifically, the difference between the maximum unsaturated output DN value and the dark offset, $D_{\mathrm{sat}}-b$, is taken as the maximum usable signal swing of the final output. The parameter $\delta_{d}$ denotes the standard deviation of repeated dark-frame DN outputs under dark conditions and is used to characterize the output noise floor. Accordingly, the ratio describes the span between the maximum usable signal swing and the output noise level of the final digital output.

Equation (8) indicates that the digital dynamic range of the final output in a single frame can be improved in two complementary ways. One is to enlarge the usable range at the bright end so that strong signals are not clipped by saturation while weak signals remain usable [22]. The other is to reduce the output noise floor so that weak signals under dark conditions can be distinguished more clearly. Guided by this idea, the present work develops a four-pixel HDR fusion method that improves both the non-saturated upper limit at the bright end and the discernibility of weak signals at the dark end.

### 2.2. Principle of the Four-Pixel HDR Fusion Method

**Figure 1:**
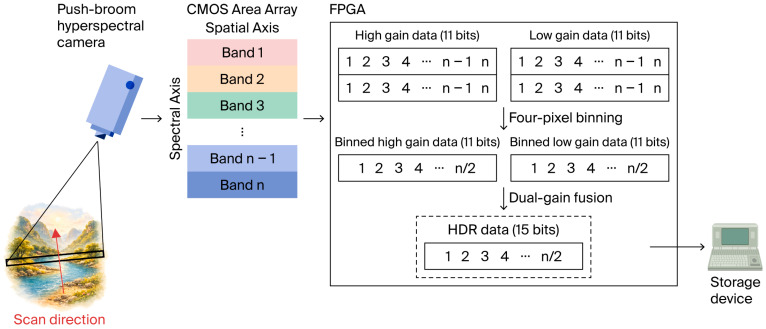
Schematic of the four-pixel HDR fusion method.

This paper proposes a four-pixel HDR fusion method tailored for the real-time acquisition system of an airborne push-broom VNIR hyperspectral camera. The schematic diagram of the method is presented in Figure 1.

Given that hyperspectral data exhibit local continuity in both the spatial and spectral dimensions [23], four-pixel binning is performed in the digital domain along the spectral and spatial dimensions for the high-gain and low-gain data streams separately. This operation aims to reduce dark noise and alleviate the computational burden of subsequent processing and transmission. The binning formula is

(9) $$D_{g}^{\prime }(u,v)=\frac{1}{4}\sum_{i=0}^{1}\sum_{j=0}^{1}D_{g}(2u-1+i,2v-1+j),\;g\in \{\mathrm{lg},\mathrm{hg}\},$$

where $D_{g}^{\prime }$ denotes the binned high-gain and low-gain data, u is the binned row index in the spectral dimension, v is the binned column index in the spatial dimension, and $D_{g}$ is the original high-gain and low-gain data.

Within the linear response region of the CMOS sensor, under constant incident illumination, the equivalent number of photoelectrons accumulated by a pixel is approximately proportional to the exposure time. Furthermore, due to the local continuity of push-broom VNIR hyperspectral data in the spatial and spectral dimensions, adjacent pixels exhibit only minor response variations. Therefore, after binning adjacent pixels, the output remains consistent with the linear model, which can be written as

(10) $$D_{g}^{\prime }=s_{g}\cdot t_{\exp}+b_{g},\;g\in \{\mathrm{lg},\mathrm{hg}\},$$

where $s_{g}$ is the conversion coefficient for high-gain and low-gain, $t_{\exp}$ is the exposure time, and $b_{g}$ is the offset for high-gain and low-gain.

Since the binned high-gain and low-gain data corresponding to the same pixel share an identical exposure time, eliminating the exposure-time term $t_{\exp}$ yields a linear relationship within the overlapping linear region:

(11) $$D_{\mathrm{hg}}^{\prime }=aD_{\mathrm{lg}}^{\prime }+o,a=\frac{s_{\mathrm{hg}}}{s_{\mathrm{lg}}},o=b_{\mathrm{hg}}-a\cdot b_{\mathrm{lg}},$$

The mapping coefficient a and offset o are employed to characterize the linear correspondence between the two gain channels. These parameters can be determined through calibration and linear regression. As shown in Equation (12), this study adopts a pixel-wise piecewise fusion strategy to generate the final HDR output. For each binned high-gain sample, a saturation check is performed. If the binned high-gain data are not saturated, they are directly output as HDR data. When the signal approaches saturation or enters the nonlinear region, the binned low-gain data, which remain within the linear response region, are used instead. In this case, the low-gain value is mapped to the high-gain equivalent domain via Equation (11) as the HDR output for that pixel.

(12) $$D_{\mathrm{hdr}}=\left\{\begin{array}{ll} D_{\mathrm{hg}}^{\prime }, & D_{\mathrm{hg}}^{\prime }\leq T_{\mathrm{sat}}\\ aD_{\mathrm{lg}}^{\prime }+o, & D_{\mathrm{hg}}^{\prime }>T_{\mathrm{sat}} \end{array}\right.,$$

where $D_{\mathrm{hdr}}$ is the HDR data and $T_{\mathrm{sat}}$ is the saturation decision threshold of the binned high-gain channel.

This fusion method involves only a threshold comparison and a single linear-mapping operation, which facilitates pipelined implementation on an FPGA and meets the demands of high-frame-rate real-time processing.

### 2.3. Calibration and Computation of Fusion Parameters

The proposed four-pixel HDR fusion involves two types of parameters: (1) the saturation decision threshold $T_{\mathrm{sat}}$ for the binned high-gain channel, used to determine the piecewise switching point, and (2) the mapping coefficient a and offset o, employed to map the binned low-gain data into the high-gain equivalent domain. Given that the linear response region of a CMOS sensor typically spans 5% to 95% of its full scale, signals exceeding this upper limit are prone to nonlinear distortion and saturation overflow. Therefore, the saturation decision threshold $T_{\mathrm{sat}}$ is set to 95% of the saturation digital value of the CMOS sensor. This threshold serves as the criterion for determining whether the binned high-gain data have approached saturation or entered the nonlinear region.

**Figure 2:**
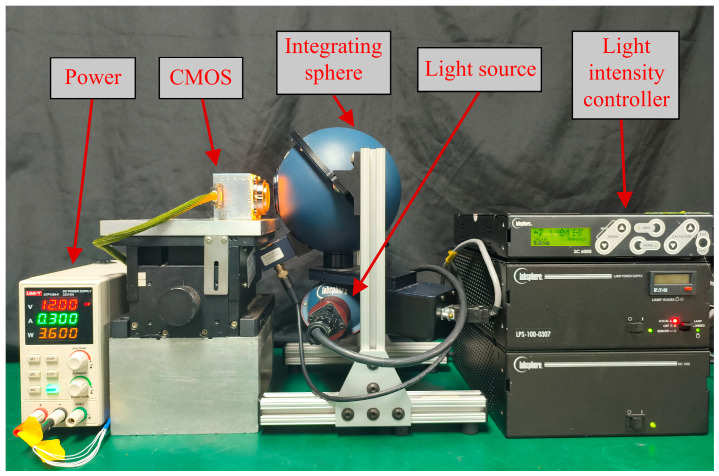
Experimental platform for fusion parameter calibration.

This paper employs an integrating sphere as a uniform light source to calibrate the mapping coefficient a and offset o. The experimental setup is illustrated in Figure 2.

During calibration, the optical power output of the integrating sphere is maintained constant, and multiple sets of raw high-gain and low-gain data are acquired by varying the exposure time. Consequently, exposure time serves as the sole variable requiring control to fit the gain relationship in the subsequent fusion process. Regulated by a high-precision FPGA counter, this exact timing control significantly improves the fitting accuracy. For each set of raw data, four-pixel binning along the spectral and spatial dimensions is performed to obtain $D_{\mathrm{hg}}^{\prime }$ and $D_{\mathrm{lg}}^{\prime }$. To minimize the influence of edge non-uniformity introduced by the integrating sphere, a stable central response region is selected within each binned image. The pixel DN values within this region are averaged to derive representative output values for each exposure time setting:

(13) $$\bar{L}=\frac{1}{N_{\Omega }}\sum_{(u,v)\in \Omega }D_{\mathrm{lg}}^{\prime }(u,v),$$

(14) $$\bar{H}=\frac{1}{N_{\Omega }}\sum_{(u,v)\in \Omega }D_{\mathrm{hg}}^{\prime }(u,v),$$

where $\bar{H}$ and $\bar{L}$ denote the average DN values within the stable region of the binned high-gain and low-gain data at the same exposure time, Ω is the set of pixels in the selected central stable region, and $N_{\Omega }$ is the number of pixels in the set Ω.

For each exposure time setting, one frame is acquired and the corresponding $\bar{H}$ and $\bar{L}$ are computed. Then, using $\left\{t_{\exp},\bar{H}\right\}$ and $\left\{t_{\exp},\bar{L}\right\}$ as samples, a first-order linear fit is performed by the least-squares method.

The system presented in this study is based on the dual-gain CMOS sensor CIS2521F (Fairchild Imaging, San Jose, CA, USA). Its key parameters are summarized in Table 1.

**Figure 3:**
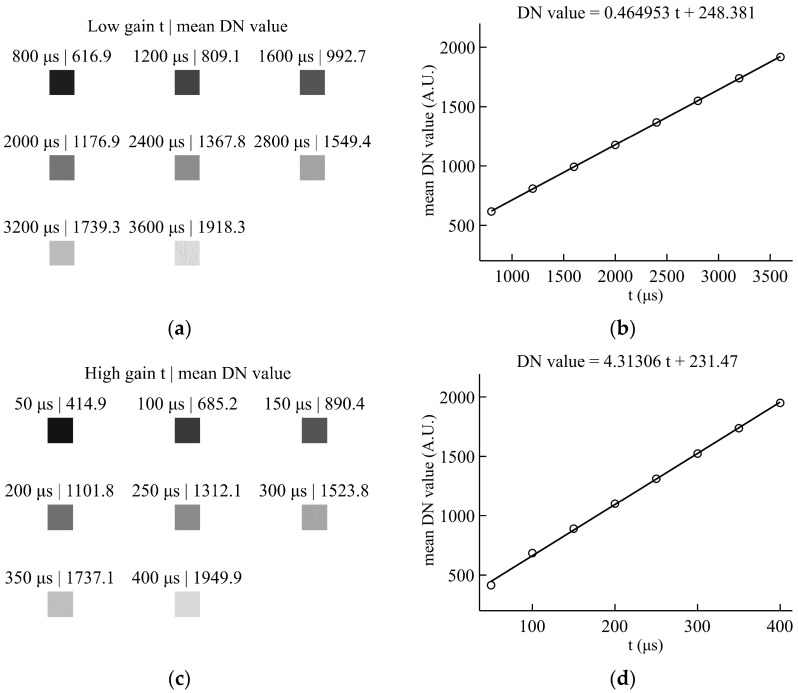
Mean DN as a function of exposure time and the corresponding linear fits. (**a**,**b**) low-gain channel; (**c**,**d**) high-gain channel.

In accordance with the aforementioned procedure, this study conducted a comprehensive optical calibration of the fusion parameters for the CIS2521F. Specifically, a 10× gain was selected for the high-gain channel, whereas a 1× gain was used for the low-gain channel, and the saturation threshold $T_{\mathrm{sat}}$ was set at 1940. A central stable region, encompassing a 100 × 100 pixel area within the binned image, was designated for calibration purposes. Subsequently, eight distinct sets of mean DN, exposure-time correspondences, derived from the linear region, were utilized as calibration samples to fit the response functions of the high-gain and low-gain channels, respectively. The fitting results for these two channels are visually presented in Figure 3.

Within the linear overlap region shared by the two channels, the mapping coefficient a and offset o are calculated from the fitting results as follows:

(15) a≈9.2766, o≈−2073.567,

Accordingly, in this implementation, the HDR fusion output formula is ultimately expressed as

(16) $$D_{\mathrm{hdr}}=\left\{\begin{array}{ll} D_{\mathrm{hg}}^{\prime }, & D_{\mathrm{hg}}^{\prime }\leq 1940\\ 9.2766\cdot D_{\mathrm{lg}}^{\prime }-2073.567, & D_{\mathrm{hg}}^{\prime }>1940 \end{array}\right.,$$

**Figure 4:**
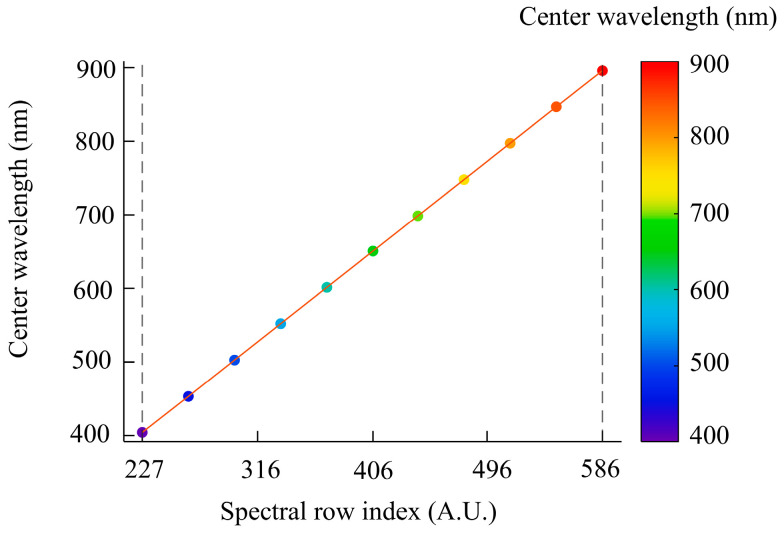
Relationship between spectral-row index and center wavelength.

After calibrating the fusion parameters, spectral calibration of the hyperspectral camera was carried out with a monochromator. This calibration established the correspondence between spectral-row index and center wavelength, from which the row-index-to-wavelength mapping in Figure 4 was obtained. The results show that the effective system response in the 400–900 nm range mainly lies between rows 227 and 586 of the spectral dimension, covering about 360 rows. To reduce redundant data and improve acquisition efficiency, the spectral-dimension region of interest (ROI) was therefore set to rows 227–586. This setting reduces the spectral readout size while still covering the effective spectral range, which in turn lowers transmission and storage burden and supports high-frame-rate acquisition.

## 3. Hardware Implementation of the System

### 3.1. Overall System Design

**Figure 5:**
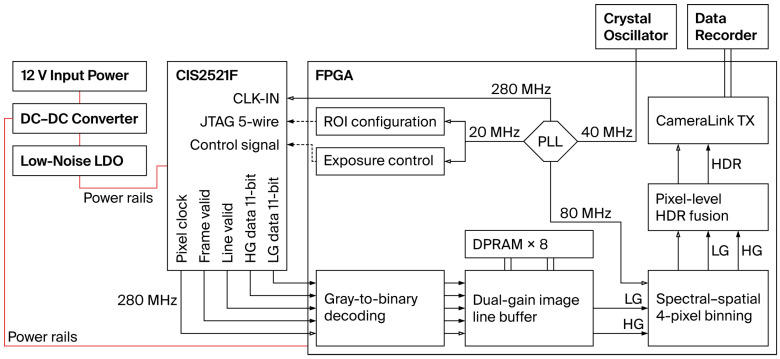
Block diagram of the HDR acquisition system.

As illustrated in Figure 5, the real-time acquisition system developed in this study is built around a CIS2521F dual-gain CMOS image sensor, utilizing a Xilinx FPGA, specifically the XC6SLX75 from the Spartan-6 series (Xilinx, San Jose, CA, USA), as the main controller. At the hardware level, the system is powered by an external 12 V supply, and the required voltage rails are generated by DC-DC converters and low-dropout linear regulators. These rails provide stable power for both the CMOS sensor and the FPGA, ensuring reliable operation. An external crystal oscillator serves as the reference clock for the FPGA, and multiple working clocks are generated through the FPGA phase-locked loop. These clocks are then distributed to the CMOS sensor and the internal FPGA modules. At the same time, the pixel clock returned by the CMOS sensor is used as the acquisition-chain clock inside the FPGA. At the control level, the FPGA configures the spectral ROI of the CMOS sensor through a low-speed JTAG interface and generates the exposure-control timing signals.

At the data-acquisition level, the raw high-gain and low-gain data are first received by the FPGA and then processed by gray-to-binary decoding, dual-gain line buffering, four-pixel binning along the spectral and spatial dimensions, and pixel-level HDR fusion. Finally, the HDR data are output to the data recorder through the Camera Link interface, enabling continuous acquisition and reliable storage under high-frame-rate conditions.

### 3.2. FPGA Implementation

**Figure 6:**
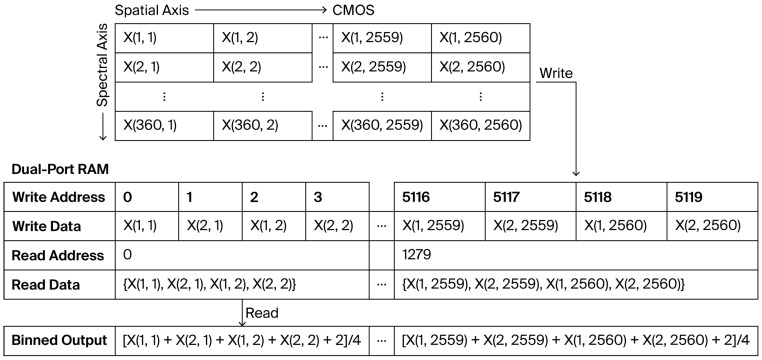
Schematic of the four-pixel binning implementation.

To implement four-pixel binning efficiently, we use a two-line buffering scheme based on dual-port RAM. As shown in Figure 6, the architecture adopts a two-line buffer with a single-readout arrangement. Two adjacent lines are buffered together, and pixels from the same column are written into the dual-port RAM with odd-even interleaved addressing. This allows the four pixel values required for binning to be fetched simultaneously in one read cycle. The four-point accumulation, shift-based division, and fixed-point rounding are then completed within the same clock cycle:

(17) $$Y=(X_{1}+X_{2}+X_{3}+X_{4}+2)>>2,$$

where Y denotes the binned pixel value and $X_{1}$, $X_{2}$, $X_{3}$, $X_{4}$ are the original DN values of four adjacent pixels.

Because the two-line buffer must be filled before the first binned output can be generated, a fixed latency occurs at the beginning of each frame. This latency can be approximated as

(18) $$t_{\mathrm{start}}\approx \frac{2N_{\mathrm{col}}}{f_{\mathrm{pix}}},$$

where $t_{\mathrm{start}}$ is the fixed per-frame buffering latency, $N_{\mathrm{col}}$ is the number of columns in a raw single line, and $f_{\mathrm{pix}}$ is the pixel clock frequency.

With $N_{\mathrm{col}}$ = 2560 and $f_{\mathrm{pix}}$ = 280 MHz in this system, the calculated $t_{\mathrm{start}}$ is approximately 18.3 μs. Once the system enters steady state operation, a ping-pong read/write mechanism is employed to prevent contention between read and write accesses. This configuration ensures that the output rate matches that of the raw CMOS data stream, thereby providing a stable and uninterrupted input for subsequent real-time processing.

**Figure 7:**
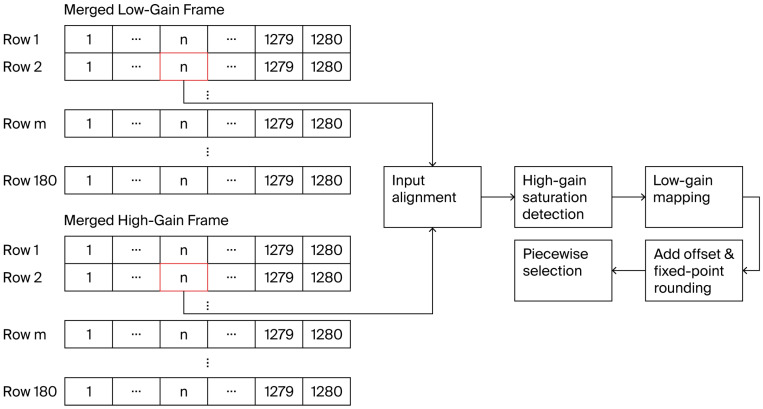
Flowchart of the dual-gain fusion pipeline.

After four-pixel binning, the FPGA carries out pixel-level HDR fusion on the binned dual-gain data. To meet the demands of high-frame-rate real-time processing, this computation is organized as a five-stage pipeline. Fusion parameters, including the mapping coefficient and offset, are pre-quantized in fixed-point form and stored in registers for online use. The five stages perform input alignment, high-gain saturation judgment, low-gain mapping multiplication, offset addition with fixed-point rounding, and piecewise output selection, respectively, as shown in Figure 7. This design supports continuous pixel-by-pixel output, and the overall pipeline latency is only five clock cycles. At a fusion-module clock of 80 MHz, the corresponding latency is 62.5 ns, which has negligible effect on the overall frame rate.

Ultimately, the proposed system achieves real-time HDR acquisition at a maximum frame rate of 290 frames/s with a single-frame resolution of 1280 × 180 pixels. The total processing latency is only 18.3 μs, accompanied by low hardware resource utilization (as shown in Table 2).

### 3.3. System Prototype

**Figure 8:**
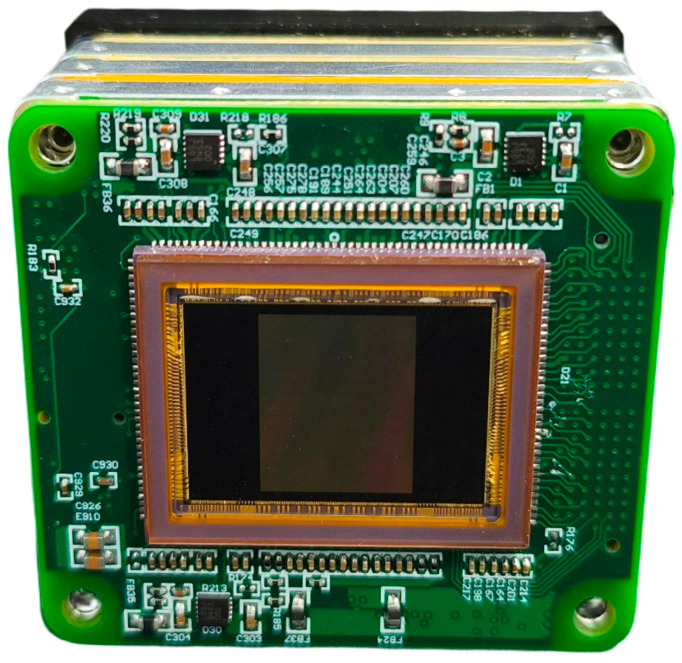
Photograph of the acquisition system hardware.

**Figure 9:**
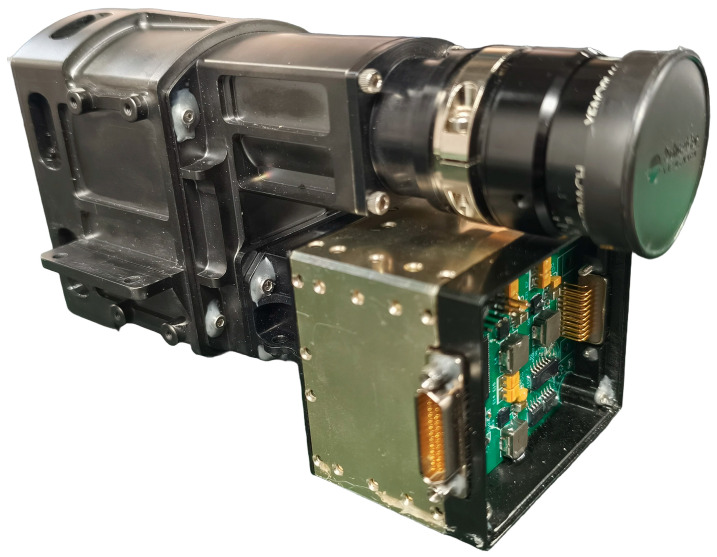
Photograph of the hyperspectral camera prototype.

Based on the proposed method, the acquisition system was designed and implemented. To achieve a compact hardware footprint, a modular architecture was adopted, utilizing FPGA Mezzanine Card (FMC) connectors to interconnect multiple circuit boards. A photograph of the acquisition system circuitry is shown in Figure 8, with overall dimensions of 5.5 cm × 5.5 cm × 3.2 cm. Figure 9 presents the integrated hyperspectral camera prototype following assembly with the spectrometer.

Furthermore, a multistage power-conversion scheme was employed to generate the required supply rails, thereby minimizing overall power consumption. Operating under a 12 V supply, the system draws an input current of approximately 0.18 A in the standby state and 0.37 A during active imaging, which corresponds to power consumptions of 2.16 W and 4.44 W, respectively. The primary physical and electrical parameters of the hardware system are summarized in Table 3.

## 4. Imaging Results and Discussion

### 4.1. Experimental Setup and HDR Imaging Results

**Figure 10:**
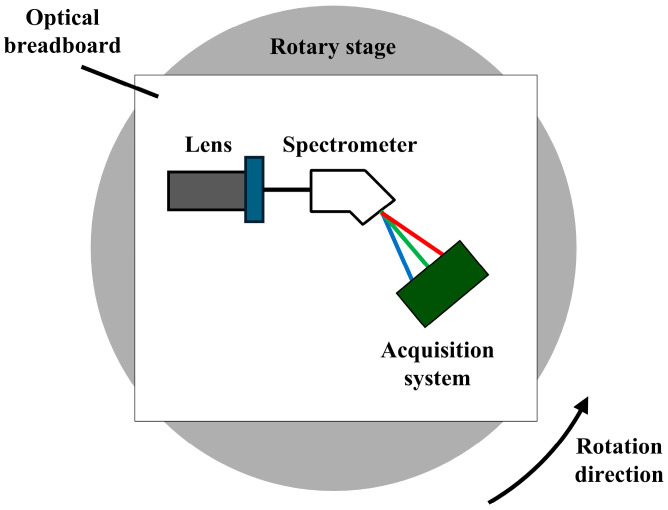
Diagram of the ground experimental apparatus.

To evaluate the performance of the designed acquisition system and demonstrate its HDR capabilities, a ground-based experimental setup was constructed utilizing a rotating platform. As illustrated in Figure 10, the hyperspectral camera, consisting of a lens, a spectrometer, and the acquisition system, is mounted on an optical breadboard secured to the rotation stage. By providing controlled relative motion between the camera and the outdoor scene, this apparatus reproduces the line-by-line scanning process required for airborne push-broom hyperspectral imaging. The selected outdoor scene, encompassing sky, vegetation, and buildings under natural illumination, guarantees the simultaneous presence of bright and dark targets within a single scan. This configuration is representative of the high-contrast conditions typically encountered in airborne VNIR applications.

**Figure 11:**
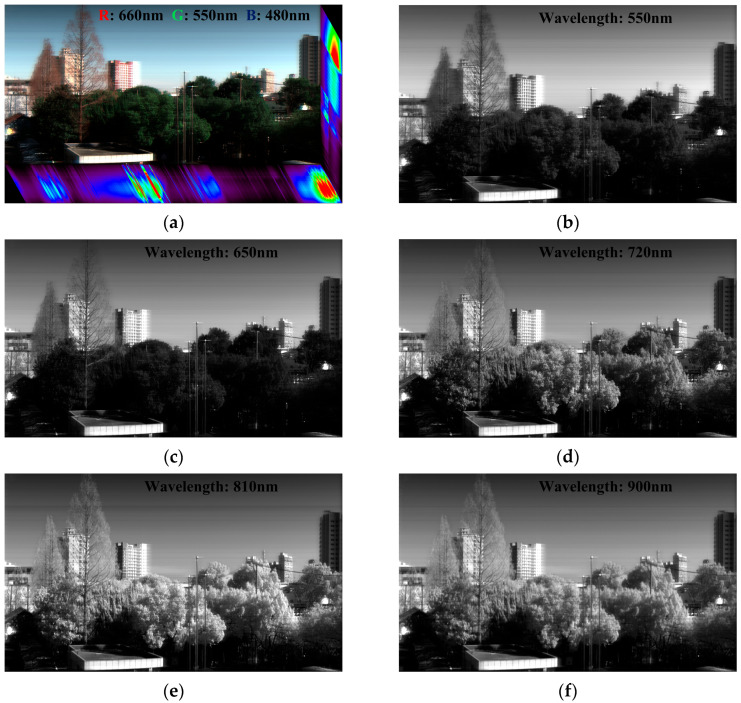
2D HDR imaging results at representative VNIR bands. (**a**) Hyperspectral data cube; (**b**) 550 nm; (**c**) 650 nm; (**d**) 720 nm; (**e**) 810 nm; (**f**) 900 nm.

Utilizing the aforementioned experimental platform, raw high-gain, raw low-gain, and fused HDR data were acquired for the same scene under identical illumination conditions. During these acquisitions, the system maintained a uniform exposure time of 5 ms to facilitate subsequent presentation and analysis. Figure 11 presents the 2D imaging results of the HDR push-broom data at several representative bands. Specifically, Figure 11a displays the HDR hyperspectral data cube, where the RGB composite image is synthesized from the 660, 550, and 480 nm bands. Meanwhile, Figure 11b–f depict the corresponding HDR images extracted at 550 nm, 650 nm, 720 nm, 810 nm, and 900 nm, respectively.

As evident from the visual results, the primary spatial structures of the scene remain clearly discernible across the entire VNIR spectrum. Representative targets, such as buildings, vegetation, and the sky, can be robustly identified at the selected wavelengths. Concurrently, the contrast between sunlit and shadowed regions is well preserved, and the spectral responses across different bands exhibit distinct variations.

**Figure 12:**
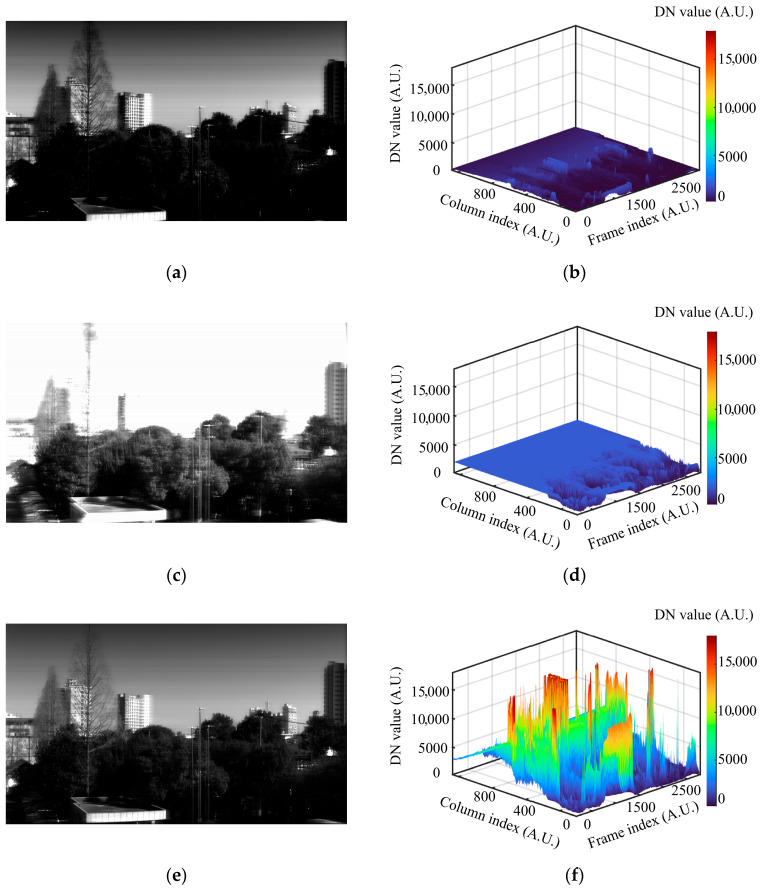
2D spatial-domain push-broom imaging results at 650 nm and 3D surface plots of the DN-value distribution. (**a**,**b**) low-gain push-broom results; (**c**,**d**) high-gain push-broom results; (**e**,**f**) HDR push-broom results.

To effectively illustrate the enhancements achieved by the HDR fusion process, the 650 nm band was selected as a representative visible wavelength for detailed analysis. Figure 12 compares the low-gain, high-gain, and fused HDR results, presenting both 2D images and the corresponding 3D surface plots of their DN value distributions. Panels (a) and (b) correspond to the low-gain push-broom results, panels (c) and (d) to the high-gain results, and panels (e) and (f) to the HDR output generated by the proposed system. As evident from the 2D images and the 3D DN-distribution surfaces, the high-gain and low-gain images suffer from missing details in highlights and shadows, respectively. In contrast, the HDR push-broom image preserves the highlight detail characteristic of the low-gain channel while retaining clearer texture information from the high-gain channel in dark regions.

**Figure 13:**
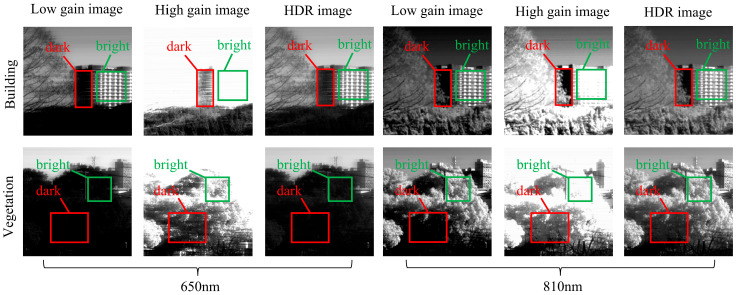
Enlarged views of typical HDR targets.

**Figure 14:**
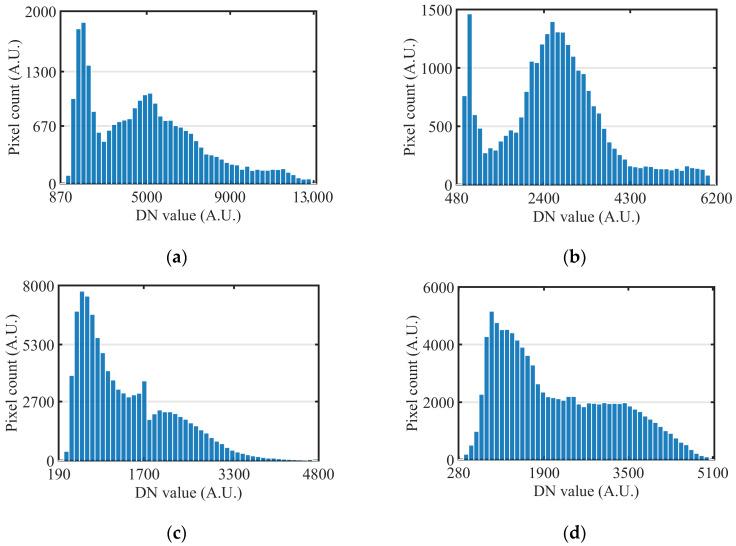
Histogram distributions of typical HDR targets. (**a**) Building at 650 nm; (**b**) Building at 810 nm; (**c**) Vegetation at 650 nm; (**d**) Vegetation at 810 nm.

Figure 13 provides enlarged views of typical high-dynamic urban targets, specifically capturing the sunlit and shadowed regions of buildings and vegetation at the 650 nm and 810 nm wavelengths. For each wavelength, the three columns correspond to the low-gain, high-gain, and fused HDR results, respectively, with the darker and brighter regions clearly marked in the images. Beyond the intuitive visual presentation, the histogram distributions of the selected HDR regions are illustrated in Figure 14. For both building and vegetation targets, the pixel responses from the sunlit and shadowed areas span a notably broader DN interval, rather than being compressed into the low-DN noise floor or accumulating near the saturation limit. This quantitative observation aligns with the visual comparisons in Figure 13, further demonstrating that the fused HDR output successfully preserves discernible responses for targets exhibiting a high brightness span within a single frame.

### 4.2. Spectral Usability Analysis

**Figure 15:**
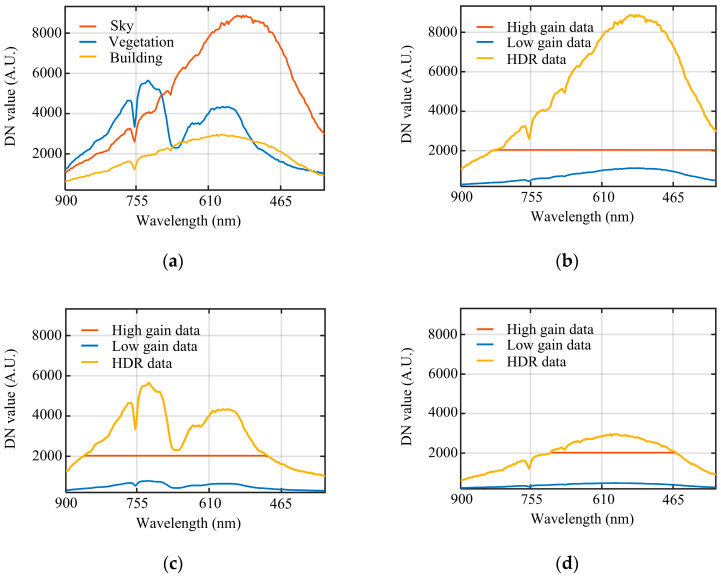
DN-value–wavelength curves for representative target pixels. (**a**) HDR curves for sky, vegetation, and buildings; (**b**) comparison of the three curves for sky; (**c**) comparison of the three curves for vegetation; (**d**) comparison of the three curves for buildings.

In hyperspectral imaging systems, data utility fundamentally depends not only on the extension of dynamic range but also on the preservation of spectral fidelity. Figure 15 further plots DN-value versus wavelength curves for representative pixels. In Figure 15a, the responses of sky, vegetation, and buildings are shown. The DN values stay continuous across the fusion transition, without obvious jumps. Figure 15b–d compare the high-gain, low-gain, and fused responses for the corresponding pixels.

For bright targets such as the sky and vegetation, the overall signal level is high, so the high-gain channel is more likely to saturate in some bands and clip local spectral features. After fusion, the HDR curve takes information from the low-gain channel in those saturated bands, which maintains continuity in the spectral variation and reduces spectral-shape distortion caused by saturation. For dark targets such as shaded buildings, the low-gain channel approaches the noise floor, making band-edge spectral differences difficult to resolve. In the HDR result, the effective signal is taken mainly from the high-gain channel, producing a clearer spectral profile and more distinguishable inter-band variation for dark targets. This improves the usefulness of the data for subsequent spectral analysis and target discrimination.

**Figure 16:**
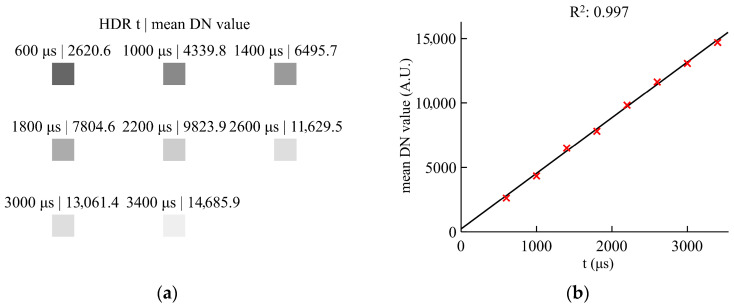
Linearity test results of the HDR data. (**a**) Representative test data samples. (**b**) Distribution of the test points and fitting results.

To more objectively demonstrate the effectiveness of the proposed fusion method in preserving spectral fidelity, eight sets of HDR samples with varying integration times were acquired under uniform illumination using an integrating sphere. The coefficient of determination, R^2^, was utilized to quantitatively evaluate the linearity of the fused HDR data. Figure 16a plots the mean DN values of these representative HDR samples, while Figure 16b illustrates their distribution relative to the extended high-gain line. The results indicate that the data maintain a highly linear distribution, with the calculated R^2^ between the samples and the high-gain fitted line reaching 0.997. This result shows that the HDR output generated by the proposed method retains good linearity within the effective operating range, thereby providing a reliable basis for the physical validity of the hyperspectral acquisition.

Furthermore, to quantitatively assess the spectral distortion introduced during the fusion process, the spectral angle mapper (SAM) metric is employed. In the field of hyperspectral imaging, SAM measures the shape similarity between a target spectrum and a reference spectrum by calculating their vector angle in a high-dimensional space. This metric determines whether two curves represent the same material, where a smaller value indicates a higher degree of similarity. In this evaluation, the unsaturated raw low-gain DN curve is utilized as the reference, and the fused HDR curve serves as the target. The quantitative results are summarized in Table 4. Specifically, the calculated average SAM values for the sky, vegetation, buildings, and the entire scene are 0.0021, 0.0056, 0.0045, and 0.0039, respectively. All metrics remain at the 10^−3^ level, indicating that the piecewise dual-gain fusion introduces limited spectral distortion within the calibrated operating range [24].

### 4.3. Ablation Study

An ablation study was conducted to evaluate six distinct processing schemes: raw low-gain, raw high-gain, low-gain with binning, high-gain with binning, dual-gain fusion without binning, and the proposed comprehensive method incorporating both binning and fusion. To quantitatively assess the digital dynamic range of the final output, an integrating sphere was utilized as a uniform illumination source to determine the maximum unsaturated DN value for each configuration. Concurrently, dark frames were acquired under strictly zero-illumination conditions to calculate the standard deviation of repeated dark-frame DN outputs.

**Figure 17:**
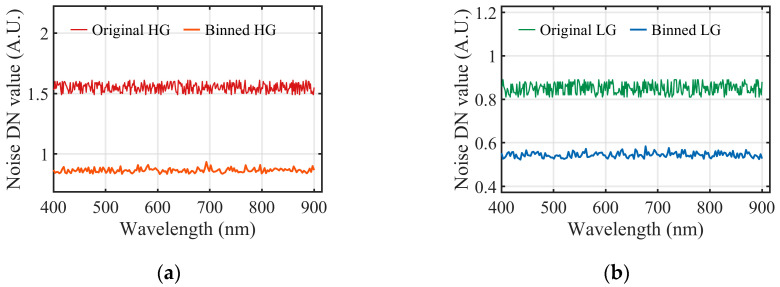
Comparison of dark-frame standard deviation across the spectral dimension. (**a**) High-gain channel; (**b**) Low-gain channel.

Figure 17 compares the standard deviation of repeated dark-frame DN outputs across the spectral dimension before and after four-pixel binning. The results reveal that the noise levels in both gain channels are significantly attenuated and stabilized following the binning operation. Specifically, in the high-gain branch, the standard deviation decreases from approximately 1.55 to 0.82. Similarly, the standard deviation in the low-gain branch drops from roughly 0.85 to 0.52.

The quantitative results of the ablation study are summarized in Table 5. Table 5 demonstrates that while utilizing pixel binning alone effectively mitigates noise, it does not alter the inherent 11-bit output ceiling of the system. Under these conditions, the digital dynamic range of the final output increases from 0.21 × 10^4^:1 to 0.35 × 10^4^:1 in the low-gain branch and from 0.10 × 10^4^:1 to 0.22 × 10^4^:1 in the high-gain branch. When dual-gain fusion is applied without pixel binning, the maximum unsaturated output DN value expands to 16,915, substantially elevating the digital dynamic range of the final output to 1.10 × 10^4^:1. This indicates that dual-gain fusion primarily serves to extend the bright-end limit. When pixel binning and dual-gain fusion are combined, the system not only preserves the expanded upper range limit but also further reduces the dark-frame standard deviation to 0.82, thereby achieving a high digital dynamic range of the final output of 2.03 × 10^4^:1. These results show that the binning and fusion operations play highly complementary roles within the proposed method.

### 4.4. Comparison and Discussion

Table 6 summarizes the key performance metrics of the proposed system alongside other representative real-time HDR CMOS acquisition systems. To provide a comprehensive comparison, the table explicitly details the output bit depth, output resolution, maximum frame rate, pixel throughput, and algorithmic latency for each system. Furthermore, to objectively evaluate the actual efficacy of the algorithms employed by the compared systems and their applicability to hyperspectral imaging, the global spectral angle mapper (SAM) of the HDR data generated by each method was evaluated under the same experimental/simulation framework. This calculation utilized the raw high-gain and low-gain data acquired from the aforementioned push-broom experiments. Correspondingly, the improvements in the digital dynamic range of the final output obtained by applying these methods to the proposed system were also estimated.

The results indicate that the proposed system achieves the lowest algorithmic latency among the compared systems. Additionally, the adopted four-pixel HDR fusion method provides the largest improvement in the digital dynamic range of the final output and the lowest global SAM value for the same scene. These results support its applicability to airborne push-broom VNIR hyperspectral cameras.

## 5. Conclusions

Airborne push-broom VNIR hyperspectral cameras face a persistent difficulty under complex illumination: they must capture highlight and shadow details within a single exposure while also maintaining a high frame rate. To address this problem, we designed a real-time HDR acquisition system based on a dual-gain CMOS sensor and developed a four-pixel HDR fusion method. The method combines spectral–spatial four-pixel binning with optically calibrated pixel-level dual-gain piecewise fusion and is implemented on an FPGA with low latency. Ground-based push-broom experiments were performed to verify the system. In a typical urban scene, the HDR results retain low-gain information in bright regions while preserving high-gain texture in dark regions, thereby reducing both highlight saturation and shadow detail loss caused by noise. The fused HDR hyperspectral data cube also maintains stable spatial–spectral relationships, with no noticeable band-to-band discontinuities or abnormal artifacts, showing that spectral fidelity is preserved while dynamic range is expanded. The spectral responses of representative target pixels remain continuous at the fusion switching points, without evident abrupt changes, and spectral analyses further showed SAM values on the order of 10^−3^ rad, indicating limited spectral distortion within the calibrated operating range, which confirms that the piecewise takeover strategy improves dynamic range while preserving spectral-shape integrity.

The experiments show that the proposed system can continuously acquire and output data at 290 frames per second, with an effective single-frame resolution of 1280 × 180 pixels. The algorithmic latency is only 0.0183 ms. The effective output bit width increases from 11 bits to 15 bits, and the digital dynamic range of the final output reaches about 2.03 × 10^4^:1, which is about 9.58 times that of the original low-gain configuration. Compared with previously reported real-time systems of similar architecture, the proposed system provides clear advantages in both frame rate and processing latency and yields a larger improvement in the digital dynamic range of the final output. These characteristics make it better suited to the engineering requirements of high-frame-rate, low-latency push-broom imaging.

However, while four-pixel binning reduces downstream data throughput, suppresses dark-end noise, and supports high-frame-rate real-time processing, it inevitably decreases the spatial and spectral sampling density of the output data. In the present system, the 2 × 2 binning performed jointly along the spatial and spectral dimensions reduces the number of samples in both dimensions to 50% of the original data. For the representative airborne VNIR tasks targeted in this work, such as water-depth retrieval and urban hyperspectral classification under high-contrast conditions, the main requirement is the simultaneous preservation of bright- and dark-end information within the same scan, i.e., avoiding highlight saturation while improving the detectability of weak signals in dark regions. For such scene-level applications, this capability is generally more important than maximizing the detectability of very small targets or extremely fine textures. Therefore, in the present application context, the sampling-density loss introduced by four-pixel binning represents a reasonable engineering trade-off for achieving lower dark-end noise, a wider usable dynamic range, and more stable real-time output. For future tasks that place greater emphasis on fine spatial structures, subtle narrow-band spectral features, or small-target detection, more flexible strategies could be adopted, such as configurable binning factors, a fusion-only mode without binning, or scene-adaptive switching between operating modes.

Furthermore, it should be noted that real-world airborne imaging environments involve several interfering factors not fully covered in the current experiments, such as temperature variations [25], platform vibrations, attitude disturbances [26], and fluctuations in external radiation conditions. Among these, temperature changes and long-term operation may cause drift in the dual-gain mapping coefficients and switching thresholds, while platform vibrations and attitude disturbances could further compromise data stability and consistency during the push-broom imaging process. Therefore, although the ground-based rotating-platform experiments in this study successfully reproduced the key geometric relationships and real-time processing constraints of push-broom imaging, the long-term robustness of the system under true airborne conditions requires further validation. Future work will focus on temperature-compensated calibration, adaptive parameter updating, and online calibration mechanisms under more realistic flight conditions. By integrating rigorous radiometric calibration with more extensive airborne field data, we aim to conduct a more systematic quantitative evaluation of the system’s dynamic range expansion capability, spectral fidelity, and engineering applicability, thereby providing a more comprehensive foundation for high-dynamic-range acquisition in real-world push-broom VNIR hyperspectral missions.

## Figures and Tables

**Table 1:** Key parameters of CIS2521F.

Parameters	Value
Native resolution	2560 × 2160 (pixels)
Number of data channels	2
High gain	×10, ×30
Low gain	×1, ×2
Data interface type	Parallel
Data type	Gray code
Output bit width	11-bit

**Table 2:** Resource utilization of the XC6SLX75 FPGA.

Slice Logic Utilization	Slice Registers	LUTs	Occupied Slices	BUFG	Block RAM	DSP48A1s
Available	93,296	46,648	11,662	16	516	132
Used	4451	4889	1952	8	70	15
Utilization	4%	10%	16%	50%	13%	11%

**Table 3:** Main physical and electrical parameters of the hardware system.

Parameters	Value
Hardware system dimensions	5.5 cm × 5.5 cm × 3.2 cm
Supply voltage	12 V
Idle current	0.18 A
Operating current	0.37 A
Idle power consumption	2.16 W
Imaging power consumption	4.44 W

**Table 4:** SAM results for representative regions.

Scene Category	SAM (Rad)
Sky	0.0021
Vegetation	0.0056
Buildings	0.0045
Full scene	0.0039

**Table 5:** Results of the ablation study.

LG	HG	Binning	Fusion	Output Bit Width (Bits)	Dark Noise (A.U.)	Maximum Unsaturated DN Value (A.U.)	Digital Dynamic Range of the Final Output (10^4^:1)
√				11	0.85	2047	0.21
	√			11	1.55	2047	0.10
√		√		11	0.52	2047	0.35
	√	√		11	0.82	2047	0.22
√	√		√	15	1.55	16,915	1.10
√	√	√	√	15	0.82	16,915	2.03

**Table 6:** Comparison of different systems.

Method	Output Bit Width (Bit)	Resolution (Pixels)	Maximum Frame Rate (Frames/s)	Pixel Throughput (Mpixel/s)	Algorithm Latency (ms)	Global SAM (Rad)	Digital Dynamic Range (10^4^:1)
This paper	15	1280 × 180	290	66.82	0.0183	0.0039	2.03
[17]	17	4096 × 4096	24	402.65	9.6	0.0128	1.07
[18]	14	2560 × 2160	50	276.48	35	0.3014	1.26

## Data Availability

The original contributions presented in this study are included in the article. Further inquiries can be directed to the corresponding author.
